# RETRACTED: Díez López, I. The Assessment of Nutritional Status in Pediatrics: New Tools and Challenges. *Children* 2023, *10*, 1151

**DOI:** 10.3390/children11010048

**Published:** 2023-12-29

**Authors:** Ignacio Díez López

**Affiliations:** 1 Pediatric Department, Basque Country University, UPV-EHU, 01009 Vitoria, Spain; ignacio.diezlopez@osakidetza.eus; 2 Pediatric Service, University Hospital of Alava, OSI Araba, Osakidetza, 01009 Vitoria, Spain

The *Children* Editorial Office retracts the article entitled “The Assessment of Nutritional Status in Pediatrics: New Tools and Challenges” by Díez Lopez [1].

Following publication, concerns were brought to the attention of the Editorial Office regarding an overlap with a previously published paper [2] from a different author group without appropriate citation. 

Adhering to our complaint procedure, an investigation was conducted by the Editorial Office and the Editorial Board, which confirmed a significant overlap with the previously published paper [2]. Given the extensive and dispersed nature of the overlap, the Editorial Office has decided to retract this Editorial [1] in accordance with the MDPI retraction policy (https://www.mdpi.com/ethics#_bookmark30, accessed on 17 December 2023) and the Committee on Publication Ethics guidelines (https://publicationethics.org/retraction-guidelines, accessed on 17 December 2023).

This retraction was approved by the Editor-in-Chief of the journal *Children*.

The authors agreed to this retraction.
